# Drug Repurposing for Targeting Cancer Stem-like Cells in Glioblastoma

**DOI:** 10.3390/cancers17182999

**Published:** 2025-09-14

**Authors:** Ana Luísa De Sousa-Coelho, Brigita Solaković, Alexandra Diogo Bento, Mónica Teotónio Fernandes

**Affiliations:** 1 Escola Superior de Saúde, Universidade do Algarve (ESSUAlg), Campus de Gambelas, 8005-139 Faro, Portugal; 2 Algarve Biomedical Center Research Institute (ABC-Ri), Campus de Gambelas, 8005-139 Faro, Portugal; 3 School of Medicine, University of Split, 21000 Split, Croatia

**Keywords:** drug repurposing, glioblastoma, glioma, glioblastoma stem-like cells, cancer stem cells

## Abstract

Glioblastoma (GBM) is one of the deadliest types of cancer, characterized by a short life expectancy after diagnosis, mostly related to therapy resistance and recurrence. GBM stem-like cells (GSCs) reside within the tumor and contribute to these features; therefore, finding drugs that specifically target such cells holds promise to halt GBM progression. The primary objective of this work is to comprehensively review and discuss the potential of hard drug repurposing to target GSCs. Several studies evaluating drugs showing anti-GSC activity, originally approved for non-cancer indications, were identified. These mainly included antidiabetics (e.g., Metformin, Phenformin, and Sitagliptin), antihypertensives (e.g., Nicardipine, Doxazosin, and Prazosin), antimicrobials (e.g., Pyrvinium pamoate, Flubendazole, and Clofazimine), and central nervous system-acting drugs (e.g., Chlorpromazine, Fluvoxamine, and Disulfiram). Relevant candidates include those that disrupt GSC metabolism, namely impairing mitochondrial function, such as Metformin, Chlorpromazine, and Pyrvinium pamoate. Multiple signaling pathways may be involved, namely the Wnt, PI3K/AKT, and STAT3 pathways, among others. Also significant were those drugs tested in combination, resulting in increased sensitivity to Temozolomide (TMZ), the standard pharmacological treatment available for GBM. Some repurposed agents, such as Disulfiram and Metformin, have already reached clinical testing, although none have yet been incorporated into clinical practice. Importantly, major translational barriers remain, like limited blood–brain barrier penetration and the lack of robust clinical trials. In conclusion, drug repurposing is an affordable and suitable strategy to target GSCs, impairing cell viability, reducing stemness, and enhancing their sensitivity to TMZ, which has potential that should be further explored to improve patients’ clinical outcomes.

## Simple Summary

Glioblastoma is one of the deadliest cancers, with most patients living only a short time after diagnosis due to treatment resistance and recurrence. A key driver of resistance is a subpopulation of tumor cells called glioblastoma stem-like cells (GSCs), which assist the tumor in resisting therapies and regrowth. This review explores the potential of using existing drugs, already approved for other diseases, to target GSCs. Several promising candidates are presented, including drugs used for treating diabetes, high blood pressure, infections, and neurological diseases, many of which can cross the blood–brain barrier. Some of these work by disrupting GSC energy production, others by blocking tumor-promoting signals, and several increase the effectiveness of current chemotherapy. By identifying safe, accessible drugs that target GSCs and improve response to treatment, this approach could open new and faster paths to improve GBM patient outcomes.

## 1. Introduction

Glioblastoma (GBM) is the most common and lethal primary brain cancer, arising from glial stem or precursor cells, as a subtype of gliomas [1]. It is characterized by rapid growth, diffuse invasion into normal brain tissue, and a dismal prognosis [2]. Despite an intensive standard-of-care therapeutic approach combining surgical resection, radiotherapy, and chemotherapy with Temozolomide (TMZ) [3], survival rates remain low, with an almost inevitable recurrence [2]. Despite this scenario, no significant therapeutic advances have been recorded in the past 20 years. Innovative approaches such as tumor-treating fields [4,5,6,7], immunotherapies [8,9], and stem cell-based therapies [10,11,12,13,14] have reached clinical trials, but clinical benefits remain limited, underscoring persistent gaps in therapeutic efficacy [15,16]. Therefore, there is an urgent need to identify new strategies to overcome the resistance of this type of cancer.

A key contributor to treatment failure is the pronounced cellular heterogeneity of GBM, which resembles the complexity of a developing organ and contains cells with stem-like properties [17]. A growing body of evidence implicates glioblastoma stem-like cells (GSCs) as key drivers of tumor progression and resistance through their capacities for self-renewal, lineage plasticity, and stress tolerance [18,19,20,21,22,23].

The existence of cancer stem cells (CSCs) at the apex of the tumor cell hierarchy or as a source of less differentiated cells in GBM tumors has been a topic of debate. In this context, a revised definition was proposed in 2024, defining GSCs as cells with long-term self-renewal capacity that generate diverse progeny, while lacking the ability to fully differentiate [24]. Recent single-cell transcriptomic studies have further revealed that GBM stemness is highly plastic, with tumor cells dynamically shifting between defined cell states in response to environmental and therapeutic pressures [23,25,26,27,28]. Whether arising from one or multiple subpopulations, GSCs are strongly associated with recurrence, therapy resistance, and clinical outcome [19,23,29,30,31], making them a critical target for effective and durable therapies for GBM [21].

One increasingly promising approach to circumvent the limitations of traditional drug discovery is drug repurposing, that is, the identification and development of new therapeutic uses for existing drugs. This strategy is especially attractive in the context of GBM, given the high attrition of novel oncologic agents and the urgent clinical need. As highlighted by Ashburn and Thor (2004), drug repurposing reduces cost, accelerates timelines, and leverages existing safety data, thereby accelerating the translational pipeline for anti-cancer therapies [32]. While much of oncology repurposing has focused on anti-cancer drugs, there is growing interest in repositioning non-cancer drugs [33,34], including antipsychotics, antidepressants [35], antimalarials [36,37], and antibiotics [38,39], that present unexpected anti-GBM activity. This focus is especially relevant because the blood–brain barrier (BBB) restricts the delivery of many systemic therapies, whereas several non-cancer drugs already exhibit favorable CNS penetration [40,41,42].

Within the domain of GSC-targeted therapy, drug repurposing has gained traction as a method to identify agents capable of selectively impairing stemness properties and sensitizing these cells to standard therapies. However, despite progress in oncology, drug repurposing for GSCs remains underdeveloped. Preclinical challenges include accurately modeling GSC biology, limited systematic testing of repurposed drugs in GSC-enrichment conditions, and additional pharmacological barriers posed by the BBB. At the mechanistic level, therapeutic strategies have focused on three major axes: (i) developmental signaling pathways (e.g., Notch, Hedgehog, Wnt) that sustain stemness; (ii) metabolic reprogramming and mitochondrial dependencies that underlie GSC survival; (iii) epigenetic regulation, which enables phenotypic plasticity and therapy evasion [21,43,44,45,46]. While each pathway offers compelling therapeutic rationale, conflicting results across studies underscore the need for integrated and comparative evaluations to identify the most effective approaches.

This review explores the landscape of drug repurposing strategies focused on targeting GSCs, with particular emphasis on the repositioning of drugs not originally approved to treat cancer. We summarize the molecular mechanisms underpinning GSC-directed therapies, review landmark and emerging repurposing studies, and assess their translational potential. Ultimately, we argue that an integrated drug-repurposing approach, especially leveraging non-cancer agents with BBB penetrance, holds promise to overcome GSC-driven therapy resistance and address the persistent challenge of GBM treatment.

## 2. Drug Repurposing in Glioblastoma

### 2.1. Soft Repurposing in Glioblastoma

“Soft repurposing” refers to the strategy of adding new cancer indications for established cancer medicines, an approach that is based on the existence of shared molecular pathways either activated or inhibited between different types of cancers [34]. Before TMZ was integrated into the first-line protocol to treat GBM [3], several other chemotherapeutic drugs were tested, like Procarbazine, Lomustine, and Vincristine [47,48,49]. Other anti-cancer targeted therapies indicated for other types of cancer also showed potential to treat GBM. Examples include Bortezomib, a proteasome inhibitor originally developed to treat multiple myeloma, Regorafenib, an inhibitor of multiple kinases originally approved for metastatic colorectal cancer (CRC) and hepatocellular carcinoma, and Bevacizumab, an anti-angiogenic agent that, although first approved for metastatic CRC, was later approved for other types of cancer by the Food and Drug Administration (FDA), including for recurrent GBM [50,51,52].

Many of these examples have been extensively reviewed elsewhere, although most of the studies have primarily focused on targeting the bulk of the tumor instead of specifically targeting glioma stem-like cells (GSCs). In summary, soft repurposing builds on drugs already validated in oncology, while hard repurposing explores drugs from non-oncologic fields, thereby offering greater novelty but also distinct translational challenges. The repurposing of drugs originally developed for conditions other than cancer has a higher potential for innovation and, therefore, will be the focus of this review.

### 2.2. Hard Repurposing in Glioblastoma

Several drugs originally developed for conditions other than cancer have shown promise when repurposed for GBM treatment, so-called “hard repurposing”. These include approved drugs used to treat diabetes mellitus, such as Metformin, antimalarial drugs such as Mefloquine, Chloroquine, and Lumefantrine, and also several drugs used for neurological diseases such as Alzheimer’s and Parkinson’s disease (Memantine and Pimavanserin, respectively), and schizophrenia or epilepsy (Fluphenazine, Fluspirilene, Valproic Acid, and Levetiracetam) [50,53]. These agents can be broadly grouped into metabolic regulators (e.g., Metformin), antiparasitic agents (e.g., Mefloquine, Chloroquine), and CNS-acting drugs (e.g., Fluphenazine, Fluspirilene, Valproic Acid, Levetiracetam). The latter group is particularly attractive because of their intrinsic BBB permeability, whereas other classes, such as antidiabetics or antimalarials, may face limitations in CNS penetration, necessitating reformulation or innovative delivery approaches. The effect of these drugs on GBM, more specifically on GSCs, was evaluated alone or in combination with TMZ and is further explored in this review.

### 2.3. Glioblastoma Models for Drug Testing

Glioblastoma stem-like cells (GSCs), a subpopulation of tumor cells with self-renewal, multipotency, and high tumorigenic potential [18], are typically enriched and studied in vitro through serum-free culture conditions that mimic aspects of the neural stem cell niche. Following surgical resection, tumor samples obtained from patients are enzymatically or mechanically dissociated into single-cell suspensions. GBM established cell lines may also be used (e.g., U87, LN229, A172, U251, T98G, and U118). These cells are then cultured in defined media supplemented with growth factors such as epidermal growth factor (EGF) and basic fibroblast growth factor (bFGF), which support the expansion of stem-like cells while suppressing differentiation, and in non-adherent conditions. Under these conditions, GSCs often form free-floating neurosphere-like structures, also named spheroids or tumor spheres, although some protocols also utilize adherent monolayer cultures on laminin-coated surfaces, for instance, to maintain their stemness and facilitate downstream analyses [54,55]. Enriched GSC cultures are typically characterized by the expression of stem cell markers such as CD133, Nestin, and SOX2, along with functional assays assessing self-renewal and tumor initiation capacity [56]. The tumorigenic potential is typically assessed using orthotopic xenograft models, in which GSCs are implanted into the brains of immunocompromised mice (e.g., NOD/SCID, NSG, and Nude mice). This approach allows researchers to evaluate the ability of GSCs to initiate and sustain tumor growth in a physiological environment that more closely mimics human GBM [19,56]. Moreover, GSC-enriched cultures may be differentiated to mimic the tumor bulk cells by introducing adhesion conditions and using FBS-supplemented culture medium (without bFGF and EGF) [55]. Altogether, the described in vitro systems, alone or in combination with xenograft in vivo models, provide a critical platform for studying GSC biology, testing therapeutic agents, and modeling GBM heterogeneity alongside resistance mechanisms [57].

## 3. Specific Targeting of Glioblastoma Stem-like Cells with Repurposed Drugs

### 3.1. Search Strategy and Selection of Studies

To comprehensively explore the strategies to target CSCs in adult GBM with hard-repurposed drugs, a systematized search protocol was defined. The search was performed in PubMed, using the following combination of predefined keywords: (“glioblastoma” OR “glioma” OR “GBM”) AND (“cancer stem cell*” OR “glioma stem cell*” OR “CSC”) and (“drug*” AND (“repurpos*” OR “reposition*”)), on 3 December 2024 (Table 1). No date restrictions were applied.

A total of 60 potentially relevant articles were identified. From these, 16 were review articles. Although related to the topic, such reviews did not comprehensively focus on hard-repurposing strategies targeting GSCs, the main goal of the current review. Only studies of adult GBM (IDH1/2–wildtype) with an explicit focus on GSCs in preclinical or translational/clinical settings that evaluated hard repurposing (drugs previously approved for non-oncologic indications) were included. Studies on pediatric GBM, IDH-mutant or non-GBM gliomas, CSC studies not specific to GBM/GSCs, and drugs already approved for any cancer indication were excluded. In total, 28 original articles met these criteria, and their data were extracted and are summarized in Table 2.

In addition, the reference lists of relevant reviews and original articles were searched to capture potential repurposed drugs targeting GSCs that may have been missed by the initial search; eligible studies identified through this process were included in the subsequent subsections.

Where clinical trial NCT identifiers are provided, they refer to records on ClinicalTrials.gov, and the trial information reflects the database status as assessed on 4 September 2025.

### 3.2. Identification of Repurposed Drugs Targeting Glioma Stem-like Cells

From the selected articles, many different drugs with the potential to target GSCs were identified (Table 2). These included drugs used for many diverse diseases, namely diabetes mellitus, hypertension, schizophrenia, depression, and infections, among other indications, which were further categorized under the Anatomical Therapeutic Classification (ATC) (Table 3). Interestingly, the most represented anatomical main groups were the “nervous system”, followed by “anti-infective for systemic use”, “alimentary tract and metabolism groups”, and “cardiovascular system”.

#### 3.2.1. Repurposed Drugs Used in Patients with Diabetes

**Figure 1:**
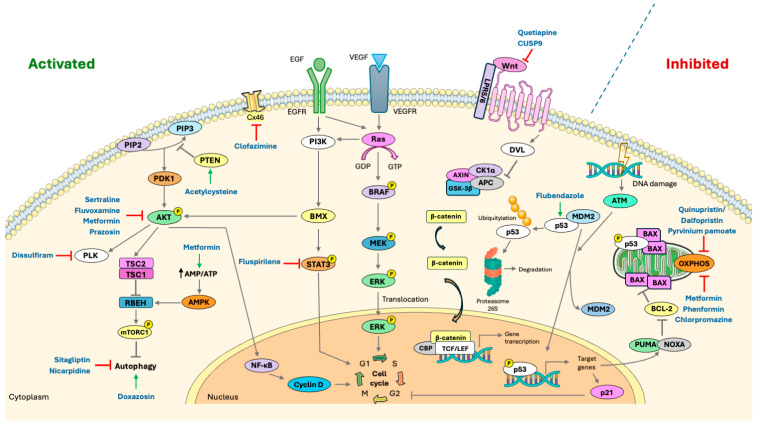
Schematic representation of key signaling pathways involved in glioblastoma pathogenesis and their modulation by repurposed drugs. Pathways frequently activated in GBM, such as PI3K/AKT, RAS/MAPK, and Wnt/β-catenin, are shown as activated, whereas tumor suppressor pathways like those mediated by p53 are commonly inactivated. Grey symbols represent signaling events: → activation or ⊣ inhibition. The black arrow represents an increased ratio (↑). Hard-repurposed compounds identified in this review are mapped to their corresponding direct or indirect targets within these pathways. The following symbols denote drug modes of action: → activation (green); ⊣ inhibition (red). Abbreviations: AKT, Protein kinase B; AMPK, AMP-activated protein kinase; APC, Adenomatous polyposis coli protein; ATM, Ataxia-telangiectasia mutated kinase; AXIN, Axis inhibition protein; BAX, Bcl-2-associated X protein; BCL-2, B-cell lymphoma 2; BMX, Bone marrow tyrosine kinase on chromosome X; BRAF, B-Raf proto-oncogene serine/threonine-protein kinase; CBP, CREB-binding protein; CK1α, Casein kinase 1 alpha; Cx46, Connexin 46; Cyclin D, G1/S-specific cyclin D; DVL, Dishevelled segment polarity protein; EGF, Epidermal growth factor; EGFR, Epidermal growth factor receptor; ERK, Extracellular signal-regulated kinase; GSK-3β, Glycogen synthase kinase-3 beta; MDM2, Mouse double minute 2 homolog; MEK, Mitogen-activated protein kinase kinase; mTORC1, Mechanistic target of rapamycin complex 1; NF-κB, Nuclear factor kappa B; OXPHOS, oxidative phosphorylation; p21, Cyclin-dependent kinase inhibitor 1 (CDKN1A); p53, Tumor protein p53; PI3K, Phosphoinositide 3-kinase; PDK1, 3-Phosphoinositide-dependent protein kinase-1; PIP2, Phosphatidylinositol 4,5-bisphosphate; PIP3, Phosphatidylinositol (3,4,5)-trisphosphate; PLK, Polo-like kinase; PTEN, Phosphatase and tensin homolog; Ras, Rat sarcoma virus protein; STAT3, Signal transducer and activator of transcription 3; TCF/LEF, T-cell factor/lymphoid enhancer-binding factor family; TSC1, Tuberous sclerosis complex 1; TSC2, Tuberous sclerosis complex 2; VEGF, Vascular endothelial growth factor; VEGFR, Vascular endothelial growth factor receptor; Wnt proteins, Wingless-related integration site proteins; β-catenin, Beta-catenin. Figure generated using Servier Medical Art (https://smart.servier.com/ accessed on 11 September 2025), licensed under CC BY 4.0 (https://creativecommons.org/licenses/by/4.0/ accessed on 11 September 2025).

Metformin is one of the most prescribed drugs for treating prediabetes and diabetes, belonging to the biguanide family of antidiabetic drugs [86]. In a research article published over a decade ago, Gritti and colleagues showed that the treatment of stem cell-enriched primary cells isolated from GBM patients with Metformin resulted in G1 phase arrest and a reduction in cell viability [85]. The authors verified that these antiproliferative effects were dependent on the functional activity of the chloride intracellular channel-1 (CLIC1) [85], a channel previously found to be involved in GBM development [87]. These findings add to a previous report in which the authors found Metformin-specific GSC-elimination was mediated by AKT signaling inactivation [88] (Figure 1). In addition, others previously showed that the survival of mice bearing an intracranial xenograft of GSC-enriched GBM cells treated with Metformin before transplantation was extended, highlighting the role of AMPK-FOXO3 activation by Metformin in GSC elimination [89]. Metformin also showed moderate cytotoxic activity in the patient-derived GBM stem cell line U3042, defined as stem-like cells and cultivated under conditions that maintained their stemness [58]. In summary, Metformin appears to act on GSCs through distinct but complementary mechanisms, which may depend on the cellular and metabolic context. While Metformin suppressed Akt phosphorylation and downstream mTOR activity, the authors found it was independent of AMPK activation [88]. However, others showed that Metformin indeed activated AMPK and its downstream effector FOXO3 [89]. Most likely independently, CLIC1 activity was inhibited by Metformin, a mechanism also noted for Phenformin [90].

Multiple clinical trials, including ongoing studies, are evaluating Metformin in GBM. A completed randomized, prospective, multicenter, controlled Phase II trial (NCT03243851) tested Metformin added to low-dose TMZ in patients with recurrent or refractory GBM and did not demonstrate clinical benefit for the combination [91].

In GSC-enriched tumor spheres that were generated from GBM primary tumors, Phenformin (another biguanide derivative) inhibited GSC proliferation and self-renewal [80]. The expression of several stemness (i.e., OCT4, SOX2, and CD44) and mesenchymal transition (i.e., YKL40 and fibronectin) markers was downregulated, while microRNA expression was altered, affecting the H19/let-7/HMGA2 pathway, in response to Phenformin treatment. Moreover, when administered to mice, Phenformin inhibited tumor growth and prolonged the overall survival of mice orthotopically transplanted with GSCs. Importantly, when combined with TMZ or dichloroacetate, a glycolysis inhibitor, Phenformin’s effects were further enhanced both in vitro and in vivo [80], highlighting its potential for GBM treatment through GSCs targeting (Figure 1).

Also for type 2 diabetes treatment, Sitagliptin is a dipeptidyl peptidase 4 (DDP4) inhibitor that increases the levels of the incretin hormones glucagon-like peptide 1 (GLP-1) and glucose-dependent insulinotropic peptide (GIP) [92]. In established GBM cell lines (U87, U251, T98G, A172, and LN229), primary cell lines, and GSCs-enriched cultures, Sitagliptin inhibited cell viability [63]. It also suppressed GSC self-renewal and stemness, showing decreased tumor sphere formation, an in vitro assay that evaluates the presence of cancer cells with stemness properties, and reduced expression of stem cell markers (i.e., CD133 and Nestin). Finally, its administration inhibited tumor growth and prolonged survival when administered to intracranial xenografted mice [63]. Interestingly, while the activity of DDP4 was decreased, the mice maintained their body weight and glycemia over time, reflecting favorable safety and tolerance [63]. Moreover, by inhibiting late autophagy, Sitagliptin was able to enhance TMZ cytotoxicity (Figure 1). Importantly, a new Phase II clinical trial (NCT07003542) that is not yet recruiting will evaluate the impact of Sitagliptin on patients with progressive grade 4 gliomas by targeting the tumor microenvironment and not the cancer cells directly.

#### 3.2.2. Repurposed Drugs Used in Patients with Hypertension

Based on the complexity of the disease and its difficult control, there are different classes of antihypertensive drugs in the market, such as diuretics, calcium channel blockers, angiotensin-converting enzyme (ACE) inhibitors, and alpha and/or beta-adrenergic receptors (AR) blockers, among others [93].

Nicardipine is a member of the calcium channel blocker class used for hypertension [94]. In patient-derived GSCs, it promoted apoptosis and sensitized cells for TMZ cytotoxicity [68]. It was reported that Nicardipine combined with TMZ inhibited autophagy and promoted apoptosis by upregulating the mTOR pathway (Figure 1). The overall survival of orthotopically xenografted mice was also prolonged when they were treated with TMZ in combination with Nicardipine [68].

Doxazosin, a long-acting α1-adrenegic receptor (α1-AR) antagonist used in the treatment of hypertension and benign prostatic hyperplasia [95], was found to enhance the anti-cancer effects of Osimertinib (an EGFR inhibitor) in a patient-derived cell line both in GSC-enrichment conditions and after differentiation, an effect mediated through autophagy induction [71] (Figure 1). This observation is important since GBM acquires resistance to Osimertinib, and Doxazosin may resensitize GBM cells to Osimertinib treatment. In another study, Doxazosin was shown to induce apoptosis and G0/G1 cell cycle arrest of the GBM LN229 and U87 MG cell lines, although its effects specifically in GCSs were not explored at that time [96].

In cells with stem-like properties isolated from patient-derived GBM tumors that were resistant to TMZ, the treatment with Prazosin (an α1-AR and α2B-AR antagonist antihypertensive drug) induced cell apoptosis, inhibited tumor growth, and prolonged the survival of orthotopic xenografted mice. In this context, Prazosin’s cytotoxic effect was independent of α1-AR and α2B-AR and relied on inhibition of the AKT pathway (Figure 1). Interestingly, dose-dependent changes in cell viability were more evident in Prazosin when compared to Doxazosin [83].

#### 3.2.3. Antimicrobial Repurposed Drugs

Cancer cells rely on both glycolysis and mitochondrial respiration for obtaining energy. Targeting GSC metabolism, which depends heavily on mitochondrial function, is a promising approach for cancer treatment [97]. Indeed, the above-mentioned biguanides (Metformin and Phenformin) are known mitochondrial complex I inhibitors [98]. Approved therapeutic agents that inhibit mitochondrial biogenesis also include antibiotics, anthelmintics, and antimycobacterial drugs, in addition to central nervous system-acting drugs, among others.

##### Antibiotics and Semi-Synthetic Derivatives

About a decade ago, erythromycins (e.g., Azithromycin), tetracyclines (e.g., Doxycycline), and glycylcyclines (Tigecycline) were evaluated in several cell lines representative of different cancers (including the GBM established cell line U87 MG), where they inhibited tumor sphere formation [84]. These findings suggested that these antibiotics could target CSCs.

In several patient-derived GBM cell lines enriched for GSCs [99], targeting mitochondrial translation with the antibiotic combination Quinupristin/Dalfopristin (Figure 1) suppressed GSC growth, affected the formation of tumor spheres, dysregulated the cell cycle, and induced apoptosis [67]. Interestingly, cells cultured in a pro-differentiation environment were less sensitive to the treatment, suggesting that Quinupristin/Dalfopristin preferentially targets undifferentiated, stem-like cells. In addition, its efficacy in disrupting OXPHOS exceeded that of TMZ, and autophagy appeared to play a compensatory pro-survival role [67].

Similarly, Minocycline, a semi-synthetic tetracycline, showed selective cytotoxic activity against GSCs, but not against the established cell line U251 [58].

Importantly, Azithromycin, Doxycycline, and Minocycline may cross the blood–brain barrier (BBB), making them more attractive for repurposing in GBM [100,101,102]. Moreover, Doxycycline is an accepted therapy for selected CNS infections and achieves therapeutically relevant CNS exposure, underscoring its translational potential [101].

##### Anthelmintics

Pyrvinium pamoate, a synthetic anthelmintic drug, inhibits mitochondrial oxidative phosphorylation [103] (Figure 1). Although it shows poor BBB penetration, studies report its potential to eliminate GSCs selectively. For instance, in two patient-derived cell lines enriched for GSCs, Pyrvinium pamoate triggered GSC-selective cytotoxic effects, whereas TMZ did not [70].

More recently, Flubendazole, from the benzimidazole class, was tested in the U251 and LN229 GBM cell lines, inducing cell death by ferroptosis accompanied by p53 and transferrin receptor (TFRC) upregulation, as well as solute carrier family 7 member 11 (SLC7A11) downregulation (Figure 1). When the same cell lines were treated under GSC-enriching conditions, Flubendazole reduced proliferation, downregulated stemness markers (i.e., CD133 and SOX2), and triggered apoptosis [60]. Although Flubendazole may cross the BBB, it is not currently approved for the treatment of CNS infections [104,105].

##### Antifungals

The azole antifungals Ketoconazole and Posaconazole increased survival in mice intracranially transplanted with patient-derived GSCs compared to untreated controls [75]. Tumors treated with either drug exhibited reduced proliferation, increased apoptosis, and decreased expression of the glycolytic enzyme hexokinase II (HK2), highlighting the potential of targeting metabolic vulnerabilities in GSCs [106]. Early-phase clinical trials evaluated whether Ketoconazole (NCT04869449) and Posaconazole (NCT04825275) could achieve therapeutically relevant concentrations in GBM tumors; however, the studies were terminated due to slow accrual.

##### Antimycobacterials

Clofazimine, mainly used for the treatment of leprosy [107], was shown in vitro to reduce GSC self-renewal and induce apoptosis in a dose-dependent manner, without affecting non-GSCs [76]. Mechanistically, it inhibited Cx46-mediated gap junction communication in GSCs, increased intracellular ROS, and synergized with TMZ. In vivo, Clofazimine reduced the growth of GSCs-derived subcutaneous tumors in mice [76]. However, its limited penetration across the BBB is a major obstacle [108], and alternative routes of administration will be needed to advance its potential clinical applicability.

#### 3.2.4. Repurposed Drugs Used in Patients with Central Nervous System Diseases

Central nervous system (CNS) drugs are a broad class of medications that primarily target the brain and spinal cord, used to treat a wide variety of neurological and psychiatric conditions. These may include those used to specifically target tumors within the CNS (such as TMZ for GBM), those used for managing cancer-related symptoms or complications [109], or those repurposed for cancer therapy. Importantly, several CNS drugs have shown potential against GSCs by disrupting mitochondrial function, blocking signaling pathways such as signal transducer and activator of transcription 3 (STAT3) and Wnt/β-catenin, or inducing cell death and differentiation, making them attractive candidates for repositioning. Their established ability to cross the BBB also enhances their translational potential, though potential clinical trials and safety profiles must be carefully considered.

##### Antipsychotics

Several typical antipsychotics used in the treatment of schizophrenia, including the phenothiazines Trifluoperazine and Chlorpromazine, as well as the diphenylbutylpiperidine derivative Fluspirilene, have demonstrated preclinical activity against GSCs.

Trifluoperazine was identified in a mitochondrial inhibitor drug screen as selectively cytotoxic to patient-derived GSCs in vitro, alongside Pyrvinium pamoate [70].

The treatment of GBM patient-derived cell lines displaying stem-like properties with Chlorpromazine inhibited GSC viability, reduced the formation of tumor spheres, and decreased the expression of stemness markers [66]. Nevertheless, GBM established cell lines growing under adhesion conditions, and non-cancer RPE-1 cells were shown to be more sensitive than GSCs (growing as tumor spheres) to treatment. The authors also found that Chlorpromazine inhibited the expression of aldehyde dehydrogenase isoform-1A3 (ALDH1A3), a stem cell marker previously proposed to contribute to increased resistance to TMZ [110]. Furthermore, in certain cells and dose combinations of Chlorpromazine and TMZ, a synergistic decrease in cell viability was observed [66]. Previous studies have also shown that Chlorpromazine inhibited the formation of tumor spheres with GSC-enrichment from a U251-derived TMZ-resistant cell line, in a dose-dependent manner. It also promoted increased survival in an orthotopic xenograft mouse model. Importantly, Chlorpromazine was shown to induce selective inhibition of mitochondrial cytochrome c oxidase (complex IV) activity [78], disrupting, like Metformin, energy metabolism (Figure 1). However, when compared with the established U251 cell line, Chlorpromazine exerted stronger cytotoxic activity in patient-derived GSCs, which was only evident for Metformin after a longer duration of treatment [58].

Chlorpromazine has been repurposed for GBM treatment in two clinical trials: an active yet incomplete Phase II trial (NCT04224441) combining Chlorpromazine with adjuvant TMZ in patients with unmethylated *MGMT* promoter (results pending), and a completed Phase I study (NCT05190315) evaluating its safety alongside standard radiotherapy and TMZ (results not yet publicly reported).

Fluspirilene significantly reduced the viability of patient-derived GSCs and decreased their ability to form tumor spheres in a dose-dependent manner [77]. In addition, it was able to reduce proliferation and invasion in both GSCs and GBM established cell lines. Its effects on GSCs were associated with the inactivation of STAT3, a key regulator of CSC maintenance (Figure 1). Moreover, Fluspirilene reduced tumor growth and prolonged the survival of mice with GSC orthotopic xenotransplants and, consistent with the in vitro results, STAT3 inhibition was detected in these tumors [77].

In contrast to typical antipsychotics, the atypical (second-generation) antipsychotics Quetiapine and Brexpiprazole, also approved for the treatment of schizophrenia, have shown potential to target GSCs through distinct mechanisms.

Quetiapine was shown to reduce proliferation and induce the preferential differentiation to the oligodendrocyte lineage of the murine GBM cell line GL261 cultured in vitro under GSC-enriching conditions, through inhibition of the Wnt/β-catenin signaling pathway [79] (Figure 1), which plays an important role in GSC proliferation [111]. Quetiapine was also reported to reduce tumor proliferation and size when GL261 cells enriched for GSCs were transplanted subcutaneously into mice and in orthotopic xenografts, increasing mouse survival. These effects were especially relevant when Quetiapine treatment was combined with TMZ [79]. Therefore, Quetiapine may suppress the growth of TMZ-resistant GBM.

Brexpiprazole was evaluated in CSCs from multiple cancer types, including the GS-Y03 patient-derived cell line [73]. While inhibiting the growth of GS-Y03, Brexpiprazole did not affect the viability of non-cancer cells. In addition, the expression of stem cell markers was reduced after the treatment of GBM cells with Brexpiprazole, as well as their sphere-forming ability. Similarly, Aripiprazole inhibited GS-Y03 cell growth and induced cell death [82].

Trifluoperazine and Chlorpromazine are commonly linked to extrapyramidal symptoms, sedation, orthostatic hypotension, and anticholinergic effects [112,113], while Fluspirilene carries similar risks and may also cause QT prolongation [114]. By contrast, Quetiapine and Brexpiprazole have a lower risk of extrapyramidal symptoms but are associated with metabolic side effects such as weight gain and dyslipidemia in the case of Quetiapine [115], or Akathisia and CYP3A4-mediated drug interactions in the case of Brexpiprazole and Aripiprazole [116]. These distinct safety profiles strongly influence their translational potential, with atypical antipsychotics generally offering a more favorable balance of efficacy and tolerability for repurposing against GSCs.

##### Other CNS Drugs

Fluvoxamine, a selective serotonin reuptake inhibitor used as an antidepressant, inhibited GSC invasion, both in vitro and in vivo, prolonging survival in orthotopic xenografts. Its mechanism involves inhibition of actin polymerization, which can be related to the suppression of both FAK (focal adhesion kinase) and AKT/mTOR signaling [81] (Figure 1).

Despite presenting moderate translational potential based on robust preclinical efficacy, Fluvoxamine use in GBM is limited by serotonin reuptake inhibitor (SSRI)-associated adverse effects and significant drug–drug interaction risks due to potent CYP1A2/2C19 inhibition [117].

The potential of Disulfiram, a drug used to treat chronic alcoholism [118], as a repurposed drug for cancer treatment was largely explored and was recently reviewed [119,120]. In both reviews, its potential for targeting not only cancer cells but also specifically CSCs is highlighted. In 2012, Disulfiram was shown to inhibit the self-renewal capacity of TMZ-resistant primary cells from GBM tumors, but it did not affect normal human astrocytes [121]. Clinical trials were then developed to evaluate its potential in GBM treatment in combination with TMZ [122,123,124]. More recently, it was described that Disulfiram regulated cell cycle distribution and decreased the clonogenic survival of GSCs [69]. The effects observed in the patient-derived GSC culture studies were independent of the inhibition of ALDH1A3, formerly considered the main mechanism for the induction of CSC toxicity by Disulfiram, and may result from alternative mechanisms, not necessarily mediated by Disulfiram itself [125]. Importantly, the indicated effects were antagonized by TMZ, discouraging the use of Disulfiram in combination with standard therapy [69]. Previous studies had shown that Disulfiram inhibited the growth of GSCs, here designated as brain tumor-initiating cells (BTIC), sensitizing these cells to TMZ treatment [126]. Moreover, treatment of mice bearing intracranial GBM transplants with Copper–Disulfiram (DSF–Cu) metal complexes and TMZ prolonged the survival of the animals [126]. Aiming to evaluate whether simultaneous ALDH and TGF-β inhibition would be effective in targeting therapeutic-resistant GBM, Liu et al. showed that combining Disulfiram with Galunisertib led to higher cytotoxicity and inhibition of tumor spheres’ growth, compared to each drug alone [127]. Finally, there is recent data showing that DSF-Cu, either in monotherapy (i.e., Disulfiram combined with Copper gluconate) or combined with tyrosine kinase receptor inhibitors, namely Dacomitinib [58], significantly reduced the viability of patient-derived GSCs. Of note, DSF-Cu shows higher cytotoxic activity when compared with Disulfiram alone. Another study reported that Disulfiram reduced the tumor-forming ability of mesenchymal GSCs, the most resistant to therapies, in a GBM orthotopic xenograft model [65].

Disulfiram, often combined with Copper, has been evaluated in several clinical trials for GBM, including both newly diagnosed and recurrent cases. Across eight registered trials, four were completed (NCT02715609, NCT01907165, NCT03034135, NCT02678975), two terminated (NCT03151772, NCT03363659), and one remains not yet recruiting (NCT01777919). Early-phase studies established maximum tolerated doses ranging from 375 to 500 mg/day, showing that Disulfiram can be safely combined with TMZ and radiotherapy [122,123,128,129]. However, while some signals of efficacy were observed in subgroups such as BRAF-mutant GBM [128], which is rare in adult GBM, the overall clinical benefit for unselected patients has been limited, with outcomes largely comparable to standard therapy alone [122,123,124,128,129].

Importantly, safety concerns have emerged, particularly in larger randomized trials. The addition of Disulfiram and Copper to chemotherapy in recurrent GBM did not improve survival but led to significantly higher rates of severe (grade ≥ 3) and serious adverse events, including hepatotoxicity and neurotoxicity [122,123,124,128,129]. These findings highlight that, despite its theoretical appeal and preclinical rationale, Disulfiram in combination with Copper is associated with substantial toxicity and has yet to demonstrate a meaningful therapeutic advantage in GBM.

Finally, Trihexyphenidyl, an anti-Parkinsonian agent, was identified in a robotic drug screen and reduced proliferation in a GSC-enriched tumor sphere model (GBM1) [72]. Nevertheless, it has limited translational potential due not only to weak preclinical evidence, but also to prominent anticholinergic toxicity including cognitive impairment and confusion in the elderly [130].

#### 3.2.5. Other Repurposed Drugs and Broader Therapeutic Strategies for Hard Repurposing in GSCs

Additional candidates for hard repurposing to target GSCs include *N*-acetylcysteine (NAC), an antioxidant approved as a mucolytic and as an antidote for paracetamol overdose, among other indications [131], and Auranofin, an oral gold compound used in the treatment of rheumatoid arthritis [132]. In one study, by depleting fumarate, NAC decreased PTEN succination, impairing GSC maintenance, as well as increasing sensitivity to both TMZ and irradiation [59] (Figure 1). In another study, Auranofin induced cytotoxicity in GSCs through a ROS-dependent mechanism, an effect that was further enhanced when p53 was silenced [61].

CUSP9, from “coordinated undermining of survival paths”, refers to a combination of nine approved non-cancer drugs, aiming to simultaneously block several signaling pathways [133,134]. The individual and concomitant use of five drugs referred to in the previously presented preclinical studies (i.e., Auranofin, Captopril, Disulfiram supplemented with Copper(II) chloride dehydrate, Minocycline, and Quetiapine) and four more (i.e., Aprepitant, Celecoxib, Itraconazole, and Sertraline), with and without TMZ, was evaluated in patient-derived GSC-enriched cell cultures [74]. CUSP9 combined with TMZ reduced GSC viability and fully abrogated tumor sphere formation, whereas the individual effects were mild. Moreover, Wnt activity was significantly reduced after treating GSCs with such a combination (Figure 1), while not being affected by any of the drugs individually. The CUSP9v3 regimen, a refined version of CUSP9, testing a combination of nine drugs targeting multiple survival pathways alongside continuous low-dose TMZ, was assessed in a clinical trial (NCT02770378), demonstrating feasibility in recurrent GBM under careful monitoring [135]. This version includes all drugs from CUSP9 but Quetiapine, which was here replaced by the antiviral drug Ritonavir.

Using a bioinformatics-based approach that analyzed differentially expressed genes (DEGs) between patient-matched primary and recurrent GBM from four public cohorts, without direct drug testing, one study validated the repurposing potential of previously investigated agents and identified additional candidates for future evaluation. The top-ranking target compounds considered to be effective against GSCs were Rosiglitazone, Nizatidine, Pantoprazole, and Tolmetin [64].

In silico screening also allows the identification of novel drug candidates for targeting GSCs. This is possible using publicly available datasets and online resources such as the Gene Expression Omnibus (GEO), The Cancer Genome Atlas (TCGA), the LINCS Data Portal, and the Connectivity Map (CMap) portal. With such a strategy, Dogra and colleagues identified different potential molecular targets in GBM, including in GSCs, with significant impact on patient survival, and candidate drugs, such as Drospirenone and Eltrombopag (used mainly as a contraceptive drug and to treat thrombocytopenia, respectively), which will need to be experimentally validated for their anti-tumor potential against GSCs [62].

## 4. Discussion

GBM remains one of the most intractable and lethal human cancers, characterized by high heterogeneity, pronounced therapeutic resistance, and poor clinical outcomes [2,136]. The concept of targeting GSCs has gained momentum due to their central role in therapy resistance, recurrence, and tumor progression [24]. In this context, drug repurposing has emerged as a particularly attractive approach, offering a cost-effective, expedited route to therapeutic innovation, especially crucial given the high attrition rate in de novo drug development [137]. This review identified multiple studies evaluating hard-repurposed drugs, i.e., medicines originally approved for non-cancer indications that have demonstrated anti-GSC activity. These include CNS-acting agents, antidiabetics, antihypertensives, and antimicrobials, among others. CNS-active drugs in particular stand out due to their inherent BBB permeability, a major pharmacokinetic hurdle in GBM treatment. Agents such as Chlorpromazine, Fluspirilene, Trifluoperazine, and Quetiapine demonstrated potent activity against GSCs in vitro and in vivo, enhancing the efficacy of TMZ in some contexts [66,70,77,79].

Nevertheless, BBB penetration remains a significant limitation. Compounds like Clofazimine and Pyrvinium pamoate, although effective against GSCs, display poor CNS bioavailability, necessitating reformulation or novel delivery strategies for clinical translation [76,84].

The unique metabolic dependencies of GSCs have become a focal point for repurposing. Several identified drugs, including Metformin, Phenformin, Sitagliptin, Fluvoxamine, Chlorpromazine, and antibiotics like Doxycycline or Tigecycline, directly or indirectly target mitochondrial function or glycolysis. This supports the emerging notion that disrupting energy metabolism can selectively affect therapy-resistant GSCs, particularly those relying on OXPHOS [63,80,84,85].

Notably, in a recent review, Tang et al. (2025) emphasized metabolic rewiring in GSCs, including amino acid metabolism [21]. While our review identified drugs targeting more canonical metabolic processes, pathways like lysine metabolism which remain underexplored in the repurposing literature suggest a promising future direction.

Single-agent therapies often fall short due to GBM’s redundancy in survival pathways. Several studies evaluated drug combinations, for instance with TMZ, showing enhanced efficacy or reversal of TMZ resistance. For example, Nicardipine, Phenformin, Chlorpromazine, and Quetiapine sensitized GSCs to TMZ, either by affecting autophagy, promoting apoptosis, or targeting stemness pathways [66,68,79,80]. However, not all combinations are synergistic. Disulfiram, for example, displayed antagonistic effects when combined with TMZ in some models [69]. This highlights the importance of preclinical validation and mechanistic understanding when designing rational combinations. Importantly, unraveling the molecular basis of these interactions, whether synergy arises from convergent pathway inhibition or antagonism results from competing stress-response mechanisms, will be critical for refining combination therapies and avoiding ineffective or counterproductive regimens.

Tang et al. identified key GSC regulatory pathways, including Notch, Wnt/β-catenin, Hedgehog, STAT3-PARN, TFPI2-JNK-STAT3, and endogenous retroviral elements like HML-2 [21]. In our review, Wnt/β-catenin and STAT3 were among the targets of repurposed agents (e.g., Quetiapine (Wnt) and Fluspirilene (STAT3)) [77,79].

Several limitations affect the comprehensiveness of the literature search, hinder the comparability of the studies, and complicate the evaluation of the translational potential of drug repurposing for targeting GSCs. First, terminological inconsistency may have complicated our systematized search. GSCs are referred to by varied terms like glioma stem cells, stem-like cells, brain tumor-initiating cells, or glioblastoma/glioma-initiating cells. Similarly, terms like “neurospheres”, “spheroids”, and “tumor spheres” are used interchangeably without standard definitions. Establishing minimal criteria for defining GSCs, such as validated stemness marker expression, functional self-renewal capacity in vitro, and tumorigenicity in orthotopic in vivo models, would improve reproducibility and comparability across studies. Second, variable GSC models are used in different studies. Several studies used established GBM cell lines (e.g., U87, LN229, and A172) and/or patient-derived GSCs, with diverse culture conditions and inconsistent validation of stemness, and not all confirmed tumorigenicity in vivo, an essential benchmark for true GSC identity. Furthermore, xenograft models are limited by their lack of immunocompetence, restricting evaluation of drug–immune interactions, which are increasingly recognized as critical in GBM biology. Third, the studies presented heterogeneous experimental designs; that is, xenograft studies varied in mouse strain, implantation site (subcutaneous vs. orthotopic), and drug administration route, affecting translational relevance. Fourth, while we aimed to focus on *IDH*-wildtype adult GBM, many studies lacked molecular or clinical annotation, raising concerns about tumor classification, especially given updates in the WHO classification [1]. Fifth, we found an underrepresentation of clinical studies, as most findings are preclinical, with few repurposed agents tested in clinical trials. Finally, we acknowledge that our initial search strategy might have missed studies in which, while indeed evaluating hard-repurposed drugs targeting GSCs, the authors did not use the term “repurposed” nor “repositioned” in their articles.

Importantly, we were able to comprehensively describe and integrate several studies that found the potential of some drugs to be repurposed for GBM treatment, many of them supporting clinical trials. Although several repurposed regimens have been, or are being, evaluated clinically, none have been formally incorporated into standard care. Contributing factors include slow accrual in this relatively uncommon disease, frequent reliance on single-arm studies with historical controls, lack of consistent benefit at diagnosis or relapse, and pharmacokinetic constraints; that is, agents that cross the BBB in other settings may still fail to achieve therapeutic concentrations within GBM tissue [128].

The intersection of GBM biology with neuroscience, the emerging field of cancer neuroscience, opens new therapeutic avenues. As Tang et al. noted, GSCs exploit neural developmental programs and immune-privileged niches to persist. The recognition that GSCs modulate immune evasion through PD-L1, TFPI2, and other cytokines suggests that repurposed immunomodulatory agents (e.g., Fluvoxamine and Disulfiram) might have dual activity. Moreover, strategies like CAR-T cells targeting GSC-specific antigens (e.g., IL13Rα2 and GRP78) represent a powerful complement to pharmacological repurposing, especially when combined with BBB-permeable agents or metabolic modulators [21].

Progress in this field will benefit from collaborative efforts that enhance methodological consistency and strengthen translational relevance. This includes the refinement of experimental models that more accurately capture the complexity of human GBM. For example, three-dimensional humanized models based on brain organoids have emerged as valuable translational platforms for studying drug repurposing [138,139]. Unlike traditional spheroid or xenograft systems, brain organoids provide a more physiologically relevant microenvironment, recapitulating human neural architecture, cellular diversity, and intercellular interactions. Such systems hold the potential to better model GSC behavior, drug penetration, and in some cases the tumor–immune interface, ultimately enabling more predictive preclinical testing of both single agents and rational drug combinations. Future translation may also benefit from early-phase, multi-center “phase 0” clinical trials to assess pharmacokinetics and CNS penetration in well-characterized patient subgroups. While no applications have yet been reported for hard repurposing specifically targeting GSCs, integration of organoid-based assays may represent a critical step forward in bridging preclinical findings with clinical translation.

Moving forward, advancing drug repurposing research for GSCs will require the adoption of clear and consistent terminology across studies, as well as the promotion of open data sharing to facilitate robust comparative analyses. In addition, closer alignment between preclinical findings and clinical endpoints is essential to bridge the gap between bench and bedside. Incorporation of next-generation omics technologies, AI-driven screening approaches, and microfluidic-based patient-derived models may further accelerate identification and prioritization of the most promising repurposed candidates. Strengthening these foundational aspects while simultaneously incorporating advanced organoid models and mechanistic studies of drug interactions will support a more cohesive research landscape and help to advance promising repurposed candidates toward meaningful clinical impact.

## 5. Conclusions

Drug repurposing offers a promising strategy to accelerate the development of effective therapies for GBM, particularly by targeting GSCs, which play a central role in treatment resistance and tumor recurrence. Evaluation of diverse drug classes, especially CNS-active agents and compounds that modulate cancer metabolism, has revealed several candidates capable of impairing GSC viability, reducing stemness, or enhancing sensitivity to TMZ. Among them, Metformin, Disulfiram, and Chlorpromazine stand out as leading candidates for clinical translation, particularly in rational combinations with TMZ and radiation. Future trials should also consider patient stratification based on GSC-associated molecular signatures to better capture treatment responsiveness.

To fully realize the potential of this approach, future progress will depend on the adoption of standardized methodologies, rigorous functional validation of GSC models, and incorporation of emerging molecular insights. Further exploration of less studied signaling pathways and integration of concepts from fields such as cancer neuroscience may reveal novel therapeutic targets. Ultimately, a coordinated, multidisciplinary effort grounded in both preclinical and clinical rigor will be key to translating repurposed drugs into durable and effective treatments for this aggressive disease.

## Figures and Tables

**Table 1:** List of terms used in the search strategy.

Cancer	Treatment	Targeted Cells
Glioblastoma	Drug	Cancer stem cells
Glioma	Repurposing or repurposed	Glioma stem cells
GBM	Repositioning or repositioned	CSC or GSC

**Table 2:** List of selected original articles using repurposed drugs to target GSCs.

Author, Year [Ref]	Repurposed Drug(s)	Main Molecular Target(s) Identified	Model(s) Used for Drug Testing
Kucinska, 2024 [58]	Metformin, Minocycline, Chlorpromazine ^1^, Disulfiram	MT-CO1 ^1^	GBM patient-derived cell line U3042
Yin, 2024 [59]	*N*-acetylcysteine	PTEN	GBM patient-derived cell lines; MGG8 and T3264 GBM cells transplanted intracranially into mice
Teng, 2024 [60]	Flubendazole	p53, TFRC, DMT1, xCT, FHC, GPX4	GBM U251 and LN229 cell lines
Jamali, 2024 [61]	Auranofin	TrxR1, AKT, p53, p21, PARP1	GBM patient-derived cell lines (OPK161, OPK257, and OPK49)
Dogra, 2024 [62]	Drospirenone, Eltrombopag	RPA3, BLVRA, PSMA2, PSMC2, HUS1	In silico screening
You, 2023 [63]	Sitagliptin	AMPK-ULK1-Beclin1 signaling pathway	Established GBM cell lines (U87, U251, T98G, A172, and LN229); GBM patient-derived cell lines (GBM−19, −23, GSC-G, -F, -Y, and -Z); U87- and patient-derived GBM cells xenografted intracranially in mice
Roddy, 2023 [64]	Rosiglitazone, Nizatidine, Pantoprazole, Tolmetin	-	Bioinformatics-based approach
Zhang, 2022 [65]	Disulfiram	USP21, FOXD1	GBM cells isolated from primary tumors or patient-derived GBM xenografts; GBM patient-derived cells implanted intracranially in mice
Matteoni, 2021 [66]	Chlorpromazine	ALDH1A3	GBM patient-derived cell lines (TS#1, TS#83, and TS#163)
Sighel, 2021 [67]	Quinupristin/Dalfopristin	OXPHOS	GBM patient-derived cell lines
Shi, 2021 [68]	Nicardipine	LC3, p62, mTOR	GBM patient-derived cell lines (SU4 and SU5); orthotopically xenografted mice
Zirjacks, 2021 [69]	Disulfiram	-	GBM patient-derived cell lines (LK7 and LK17)
Datta, 2021 [70]	Trifluoperazine, Pyrvinium pamoate	-	GBM patient-derived cell lines (0827 and 0923)
Suzuki, 2020 [71]	Doxazosin	LC3, p62	GBM patient-derived cell line (GS-Y01)
Vargas-Toscano, 2020 [72]	Trihexyphenidyl	-	GBM cell line HSR-GBM1 (GBM1)
Suzuki, 2019 [73]	Brexpiprazole	-	GBM patient-derived cell line (GSY03)
Skaga, 2019 [74]	Aprepitant, Auranofin, Captopril, Celecoxib, Disulfiram, Itraconazole, Minocycline, Quetiapine, Sertraline	Wnt activity	GBM patient-derived cell lines
Agnihotri, 2019 [75]	Ketoconazole, Posaconazole	HK2	GBM patient-derived cell lines (GSC8–18, GSC7–2, GBM8, and GSC30); mice intracranially transplanted with GSCs
Mulkearns-Hubert, 2019 [76]	Clofazimine	Cx46	Established GBM xenografts T4121, T3691, and T387; mice transplanted subcutaneously with GBM cells
Dong, 2017 [77]	Fluspirilene	STAT3	GBM patient-derived cell lines (TGS01, TGS04, and KGS01); intracranial transplantation of TGS04 cells in mice
Oliva, 2017 [78]	Chlorpromazine	MT-COX activity	TMZ-sensitive U251 cells and TMZ-resistant cells derived from U251 cells (UTMZ); patient-derived GBM xenograft cell lines (J × 12, J × 39)
Wang, 2017 [79]	Quetiapine	Wnt/β-catenin signaling pathway	Murine GBM cell line GL261; GL261 cells transplanted subcutaneously into mice and in orthotopic xenografts
Jiang, 2016 [80]	Phenformin	HMGA2	GBM patient-derived cell lines (HF2414, HF2355, HF2354, and HF2587); mice orthotopically transplanted with GBM cells
Hayashi, 2016 [81]	Fluvoxamine	FAK and AKT/mTOR signaling	GBM patient-derived cell lines; orthotopically xenografted mice
Suzuki, 2016 [82]	Aripiprazole	-	GBM patient-derived cell line (GS-Y03)
Assad Kahn, 2016 [83]	Prazosin	AKT pathway	GBM patient-derived cell lines (TG1, TG16, GBM5, and GBM44)
Lamb, 2015 [84]	Doxycycline, Azithromycin, Tigecycline, Pyrvinium pamoate	-	GBM cell line U87
Gritti, 2014 [85]	Metformin	CLIC1	GBM patient-derived cells

^1^ Molecular target reported for Chlorpromazine only. Abbreviations: AKT, serine/threonine-protein kinase (Protein kinase B (PKB)); ALDH1A3, Aldehyde dehydrogenase 1 family member A3; AMPK, AMP-activated protein kinase catalytic subunit alpha-1; BECN1, Beclin-1; BLVRA, Biliverdin reductase A; CLIC1, Chloride intracellular channel protein 1; CTNNB1, Beta-catenin; DMT1, Divalent metal transporter 1 (SLC11A2); FAK, Focal adhesion kinase; FHC, Ferritin heavy chain; FOXD1, Forkhead box D1; Cx46, Connexin 46; GPX4, Glutathione peroxidase 4; HK2, Hexokinase II; HUS1, HUS1 checkpoint homolog; LC3, Microtubule-associated protein 1A/1B light chain 3; MT-CO1, Cytochrome c oxidase subunit I; MT-COX, Mitochondrial cytochrome c oxidase activity (complex IV); mTOR, Mechanistic target of rapamycin; OXPHOS, Oxidative phosphorylation complexes; p53, Tumor protein p53; PARP1, Poly [ADP-ribose] polymerase 1; PSMA2, Proteasome subunit alpha type-2; PSMC2, Proteasome 26S ATPase subunit 2; PTEN, Phosphatase and tensin homolog; RPA3, Replication protein A 3; p62, Sequestosome-1 (SQSTM1); STAT3, Signal transducer and activator of transcription 3; TrxR1, Thioredoxin reductase 1 (TXNRD1); USP21, Ubiquitin-specific peptidase 21; xCT, Cystine/glutamate transporter (SLC7A11).

**Table 3:** Repurposed drugs targeting glioma stem cells classified by ATC.

Drug	ATC Code ^1^
A—Alimentary tract and metabolism	
Aprepitant	A04AD12
Metformin	A10BA02
Nizatidine	A02BA04
Pantoprazole	A02BC02
Phenformin	A10BA01
Rosiglitazone	A10BD04
Sitagliptin	A10BH01
B—Blood and Blood-Forming Organs	
Eltrombopag	B02BX05
C—Cardiovascular system	
Captopril	C09AA01
Doxazosin	C02CA04
Nicardipine	C08CA04
Prazosin	C02CA01
G—Genito-urinary system and sex hormones	
Drospirenone	G03AA12
J—Anti-infective for systemic use	
Azithromycin	J01FA10
Clofazimine	J04BA01
Doxycycline	J01AA02
Itraconazole	J02AC02
Ketoconazole	J02AB02
Minocycline	J01AA08
Posaconazole	J02AC04
Quinupristin/Dalfopristin	J01FG02
Tigecycline	J01AA12
M—Musculo-skeletal system	
Auranofin	M01CB03
Celecoxib	M01AH01
Tolmetin	M01AB03
N—Nervous system	
Aripiprazole	N05AX12
Brexpiprazole	N05AX16
Chlorpromazine	N05AA01
Disulfiram	N07BB01
Fluspirilene	N05AG01
Fluvoxamine	N06AB08
Quetiapine	N05AH04
Sertraline	N06AB06
Trifluoperazine	N05AB06
Trihexyphenidyl	N04AA01
P—Antiparasitic products, insecticides, and repellents	
Flubendazole	P02CA05
Pyrvinium pamoate	P02CX01
R—Respiratory system	
Acetylcysteine	R05CB01

^1^ ATC, Anatomical Therapeutic Chemical system. Only one ATC code is provided per drug, corresponding to either its first approved indication or its most recognized therapeutic use.
